# Survival and risk factors among upper tract urothelial carcinoma patients after radical nephroureterectomy in Northeast China

**DOI:** 10.3389/fonc.2022.1012292

**Published:** 2022-10-25

**Authors:** Jianing Gao, Jingya Liu, Jianyu Liu, Shiyan Lin, Dexin Ding

**Affiliations:** ^1^ Department of Urology Surgery, Harbin Medical University Cancer Hospital, Harbin, China; ^2^ Department of Breast Surgery, Harbin Medical University Cancer Hospital, Harbin, China; ^3^ Department of Respiratory Medicine, Harbin Medical University Cancer Hospital, Harbin, China; ^4^ Department of Anesthesiology, Harbin Medical University Cancer Hospital, Harbin, China

**Keywords:** radical nephroureterectomy (RNU), upper tract urothelial carcinoma (UTUC), recurrence, metastasis, prognosis

## Abstract

**Objective:**

The study objective was to investigate the prognostic risk factors related to overall survival (OS), cancer-specific survival (CSS), recurrence-free survival (RFS), and metastasis-free survival (MFS) after radical nephroureterectomy (RNU) for upper tract urothelial carcinoma (UTUC). Patients were then divided into different risk groups (based on their number of prognostic risk factors), and specific postoperative treatment plans were formulated for patients in different risk groups.

**Methods:**

We retrospectively analyzed the data of 401 patients with UTUC who underwent RNU between 2010 and 2020. Univariate and multivariate Cox regression analyses were used to evaluate the associations of clinicopathological variables with prognosis among UTUC patients. Kaplan–Meier survival analysis of patients in different risk groups (based on their number of prognostic risk factors) was conducted.

**Results:**

Multivariate Cox regression analysis showed that sex (being male), LVI, pT stage (>pT2), and lack of postoperative intravesical instillation were independent risk predictors of shorter OS, CSS, RFS, and MFS (all P<0.05). Laparoscopic RNU was also associated with shorter OS, CSS, and MFS, but not with shorter RFS (P=0.068). After risk stratification, the 5-year OS, CSS, RFS, and MFS in the high-risk group were 42.3%, 46.4%, 41%, and 46%, respectively.

**Conclusions:**

Sex (being male), LVI, pT stage (>pT2), and intravesical instillation were independent predictors of OS, CSS, RFS, and MFS for UTUC. All were risk factors, except for intravesical instillation, which was a protective factor. Additionally, laparoscopic RNU was an independent risk factor for OS, CSS, and MFS. Patients in the high-risk group may benefit greatly from adjuvant or neoadjuvant chemotherapy.

## Introduction

Upper tract urothelial carcinoma (UTUC) is a rare malignant tumor, accounting for about 5–10% of all urothelial carcinomas, with poor prognosis (1, 2). Radical nephroureterectomy (RNU) and bladder cuff resection represent the gold standard for the treatment of non-metastatic UTUC (3), and postoperative adjuvant chemotherapy (AC) is the main treatment for postoperative recurrence and distant metastasis in patients with UTUC (4).

In the past few years, the UTUC diagnostic and resection accuracy have been improved, but the survival outcomes of UTUC patients have not significantly changed (5), and the high postoperative recurrence rate and distant metastasis rate pose heavy economic and psychological burdens on UTUC patients. Therefore, it is important to identify the relevant factors that influence UTUC survival outcomes. The prognosis of UTUC patients varies greatly and is obviously individualized. It has been reported that factors associated with poor prognosis among UTUC patients after RNU include being male, history of smoking, pathologic tumor (pT) stage, tumor grade, tumor location, lymphatic vascular invasion (LVI), hydronephrosis, tumor multifocality, ureteroscopy, history of transurethral resection of bladder tumor (TURBT), and surgery type (6–15). However, there is little research that comprehensively shows the relationships between the prognostic factors and overall survival (OS), cancer-specific survival (CSS), recurrence-free survival (RFS), and metastasis free survival (MFS).

Thus, we aimed to assess the role of a large number of clinicopathological variables in predicting OS, CSS, RFS, and MFS in a long follow-up study of UTUC patients after RNU. We then aimed to stratify the patients based on the identified prognostic factors in order to identify risk groups. The high-risk patients would be more likely to benefit from AC and/or neoadjuvant chemotherapy (NAC), while low-risk patients would benefit from avoiding the risk of treatment-related adverse events.

## Methods

### Patient selection

We conducted a detailed retrospective review of the demographic and clinicopathological data of 401 patients who had undergone RNU during a 10-year period between 2010 and 2020 and then had been followed-up (median follow-up duration: 44.7 months; range: 2 to 140 months). The exclusion criteria were partial ureterectomy, radiotherapy or neoadjuvant chemotherapy (NAC) prior to surgery, lack of relevant data, loss to follow-up, and co-existing cancer types. All patients signed informed consent forms at the time of admission.

### Surgery and AC

All patients with UTUC underwent open or laparoscopic RNU (ORNU or LRNU) according to standard procedures, which included extrapital dissection of the kidney and removal of the entire ureter length and adjacent portions of the bladder cuff. Lymphadenectomy was performed if preoperative imaging reports showed suspicious lymph node (LN) status or if positive LNs were found during surgery. AC is usually recommended for patients with pathological LN metastasis, T3–T4 stage, and positive surgical margins.

### Data collection and evaluation

Preoperative variables included sex, age, body mass index (BMI), history of smoking, family history of cancer, history of TURBT, and preoperative hydronephrosis. Intraoperative data included surgical method (ORNU or LRNU). Postoperative data included whether AC was given and whether intravesical instillation (gemcitabine or pirarubicin) was performed. Pathological data included pathological stage, maximum tumor diameter, lateral tumor location (renal pelvis and/or ureter), tumor grade, lymphovascular invasion (LVI), tumor multifocality, tumor necrosis (TN), and LN status.

### Follow-up

Follow-up began after discharge, generally at least every 3–4 months for the first year, every 6 months for the second year, and at least once a year thereafter, according to National Comprehensive Cancer Network (NCCN) guidelines. Follow-up methods included telephone and outpatient follow-up. Follow-up examinations included cystoscopy, urine cytology, physical examination, routine blood analysis, blood biochemistry analysis, computed tomography (CT) scans, and chest X-rays. History of smoking was defined still smoking at the time of diagnosis (usually for ≥5 years) or previously smoked for a long time (≥10 years) but has quit smoking for <1 year. Family history of cancer was defined as a history of cancer in an immediate family member (including a parent or sibling) of the patient. Tumor multifocality was defined as the simultaneous presence of two or more pathologically confirmed tumors in any upper urinary tract location. Tumor grade and stage were determined based on the 2017 American Joint Committee on Cancer (AJCC) tumor, node, metastatic (TNM) stage and the 2004 World Health Organization (WHO) tumor classification. Overall survival (OS) was defined as the time from the day of RNU to death. Cancer-specific survival (CSS) was defined as the time from the day of RNU to death due to UTUC. Recurrence-free survival (RFS) was defined as the time from the day of RNU to local or distant recurrence (urethral, intravesical, contralateral renal pelvis, and/or ureter recurrence). Metastasis-free survival (MFS) was defined as the time from the day of the RNU to the occurrence of metastasis (e.g., bone, liver, lung or metastasis). The low-risk group was defined as having 0 or 1 independent risk factors, the medium-risk group was defined as having 2 independent risk factors, and the high-risk group was defined as having 3–5 independent risk factors.

### Statistical analysis

All analyses were performed using IBM SPSS Statistics v26.0 (IBM, Armonk, New York, USA) or GraphPad Prism v8.0.2(GraphPad Software Inc.). All continuous variables are reported as median values and ranges. Univariate and multivariate Cox regression analyses were used to evaluate the relationships of clinicopathological variables with OS, CSS, RFS and MFS. Kaplan–Meier analysis was used for survival analysis. P<0.05 was considered statistically significant.

## Results

**Table 1** lists the clinicopathological features of the 401 patients. The median age of the UTUC patients in our cohort was 67 years (range from 33 to 90) and the median follow-up duration was 44.7 months (range from 2 to 140). 206 (51.4%) patients were male and 167 (41.6%) underwent ORNU rather than LRNU. 183 (45.6%) patients underwent intravesical instillation after surgery. 45 (11.2%) patients were positive for LVI. There were 128 (31.9%) patients at Tis, Ta, and T1 stage, 87 (21.7%) at T2 stage, and 186 (46.4%) at T3–T4 stage. During the follow-up, 130 patients (32.4%) had local recurrence and 100 (24.9%) had lymphatic spread and/or distant metastasis. 91 patients (22.7%) died from UTUC and 115 (28.7%) died from all causes.

**Table 1:** Clinicopathologic features for the overall cohort of 401 patients with UTUC after RNU.

Variables	All patients 401 (%)
Age at UTUC (years), median (range)	67 (33-90)
Follow-up (months), median (range)	44.7 (2-140)
Sex, Male	206 (51.4)
BMI≥30 (kg/m2)	31 (7.7)
The history of smoking, yes	129 (32.2)
The history of family cancer, yes	30 (7.5)
The history of TURBT, yes	79 (19.7)
Adjuvant chemotherapy, yes	104 (25.9)
Intravesical instillation, yes	183 (45.6)
Surgery approach, Open	167 (41.6)
Recurrence	130 (32.4)
Metastasis	100 (24.9)
Die from UTUC	91 (22.7)
LVI, yes	45 (11.2)
Tumor grade, High	378 (94.3)
Tumor size ≥2cm	317 (79.1)
Tumor side, Left	196 (48.9)
Hydronephrosis, yes	292 (72.8)
Tumor necrosis, yes	11 (2.7)
Multifocality, yes	86 (21.4)
Tumor location	
Ureteral	170 (42.4)
Renal pelvis	200 (49.9)
Both	31 (7.7)
pT-stage	
Tis Ta T1	128 (31.9)
T2	87 (21.7)
T3-T4	186 (46.4)
pN-stage, N+	37 (9.2)

UTUC, upper tract urothelial carcinoma; RNU, radical nephroureterectomy; LVI, lymphatic vascular infiltration.

Primarily, we analyzed the prognostic value of multiple clinicopathological variables for OS, CSS, RFS, and MFS. First, univariate Cox regression analyses showed that sex, history of TURB, postoperative AC, postoperative intravesical instillation, surgery type, LVI, hydronephrosis, tumor multifocality, tumor location, pT stage, and pN stage were correlated with shorter OS, CSS, RFS, and MFS (**Table 2**, all P<0.05). Univariate Cox regression analyses also showed that TN was associated with shorter RFS and MFS, but not with OS or CSS.

**Table 2:** Univariate Cox proportional hazard regression analysis of the risk factors for UTUC.

Variables	OS		CSS		RFS		MFS	
	*HR (95% CI)*	*p*	*HR (95% CI)*	*p*	*HR (95% CI)*	*p*	*HR (95% CI)*	*p*
Age≥67(years)	1.41 (0.97, 2.05)	0.063	1.17 (0.77, 1.77)	0.441	0.96 (0.68, 1.36)	0.858	1.06 (0.71, 1.56)	0.772
Sex, Male	1.53 (1.05, 2.21)	0.026	2.10 (1.36, 3.24)	0.001	1.66 (1.17, 2.36)	0.005	1.88 (1.25, 2.84)	0.002
BMI≥30 (kg/m2)	0.94 (0.46, 1.93)	0.873	1.05 (0.48, 2.27)	0.896	1.14 (0.61, 2.12)	0.664-	1.06 (0.51, 2.18)	0.874
The history of smoking, yes	0.95 (0.64, 1.41)	0.804	1.12 (0.73, 1.72)	0.609	0.97 (0.67, 1.40)	0.876	0.97 (0.67, 1.40)	0.942
The history of family cancer, yes	1.19 (0.62, 2.29)	0.592	1.36 (0.69, 2.72)	0.376	1.68 (0.98, 2.88)	0.057-	1.43 (0.74, 2.74)	0.284
The history of TURBT, yes	2.01 (1.36, 2.97)	0.000	2.09 (1.35, 3.32)	0.001	2.16 (1.49, 3.12)	0.000	2.18 (1.44, 3.29)	0.000
Adjuvant chemotherapy, yes	0.65 (0.44, 0.95)	0.029	0.65 (0.42, 1.00)	0.05	1.60 (1.12, 2.31)	0.01	1.89 (1.26, 2.83)	0.002
Intravesical instillation, yes	0.47 (0.32, 0.70)	0.000	0.47 (0.30, 0.74)	0.001	2.22 (1.36, 3.65)	0.007	0.54 (0.36, 0.82)	0.004
Surgery approach, LRNU	2.41 (1.64, 3.53)	0.000	2.37 (1.55, 3.630	0.000	1.83 (1.30, 2.59)	0.001	2.38 (1.59, 3.57)	0.000
LVI, yes	8.20 (5.45, 12.32)	0.000	9.59 (6.17, 14.9)	0.000	6.09 (4.10, 9.06)	0.000	8.39 (5.47, 12.86)	0.000
Tumor grade, High	1.25 (0.54, 2.84)	0.595	1.51 (0.55, 4.13)	0.415	1.16 (0.54, 2.50)	0.687	1.29 (0.52, 3.18)	0.573
Tumor size ≥2cm	1.61 (0.96, 2.69)	0.07	1.41 (0.81, 2.46)	0.22	1.60 (0.99, 2.58)-	0.051	1.61 (0.93, 2.79)	0.087
Tumor side, Left	1.04 (0.72, 1.50)	0.849	0.88 (0.58, 1.33)	0.538	0.90 (0.63, 1.27)	0.559	1.01 (0.68, 1.50)	0.938
Hydronephrosis, yes	2.26 (1.35, 3.79)	0.002	2.91 (1.55, 5.46)	0.001	2.27 (1.41, 3.66)	0.001	2.28 (1.41, 3.67)	0.000
Tumor necrosis, yes	2.82 (1.23, 6.43)	0.014	2.21 (0.81, 6.04)	0.122	2.63 (1.22, 5.64)	0.013	3.11 (1.36, 7.13)	0.007
Multifocality, yes	2.25 (1.54, 3.30)	0.000	2.51 (1.65, 3.84)	0.000	2.70 (1.89, 3.86)	0.000	2.57 (1.72, 3.86)	0.000
Tumor location		0.004		0.001		0.000		0.001
Ureteral vs. Renal pelvis	1.46 (0.99, 2.17)	0.06	1.57 (1.00, 2.47)	0.049	1.31 (0.90, 1.90)	0.153	1.45 (0.94, 2.22)	0.088
Both vs. Renal pelvis	2.66 (1.48, 4.76)	0.001	3.34 (1.79, 6.22)	0.000	3.03 (1.79, 5.13)	0.000	3.15 (1.73, 5.72)	0.000
pT-stage		0.000		0. 000		0.000		0.000
T2 vs. Tis Ta T1	2.92 (1.44, 5.92)	0.003	5.29 (1.94, 14.47)	0.001	2.60 (1.45, 4.64)	0.001	5.08 (2.02, 12.74)	0.001
T3-T4 vs. Tis Ta T1	7.11 (3.87, 13.08)	0.001	14.3 (5.74, 35.4)	0.000	4.14 (2.51, 6.84)	0.000	12.48 (5.42, 28.73)	0.000
pN-stage, N+	8.32 (5.41, 12.81)	0.000	9.27 (5.82, 14.75)	0.000	5.93 (3.87, 9.10)	0.000	8.13 (5.20, 12.84)	0.000

UTUC, upper tract urothelial carcinoma; RNU, radical nephroureterectomy; LVI, lymphatic vascular infiltration; LRNU, laparoscopic RNU.

The clinicopathological variables with significant association (P<0.05) with OS, CSS, RFS, and MFS were used in the multivariate Cox regression analyses of OS, CSS, RFS, and MFS, respectively. The multivariate Cox regression analyses showed that sex (male), postoperative intravesical instillation, LVI, and pT stage (>pT2) were independent risk predictors of shorter OS, CSS, RFS, and MFS (**Table 3**, all P<0.05). LRNU was associated with shorter OS, CSS, and MFS, but not with shorter RFS (P=0.068).

**Table 3:** Multivariate Cox proportional hazard regression analysis of the risk factors of UTUC.

Variables	OS		CSS		RFS		MFS	
	*HR (95% CI)*	*p*	*HR (95% CI)*	*p*	*HR (95% CI)*	*p*	*HR (95% CI)*	*p*
Sex, Male	1.52 (1.02, 2.27)	0.037	2.10 (1.31, 3.35)	0.002	1.51 (1.04, 2.20)	0.028	1.88 (1.21, 2.91)	0.005
The history of TURBT, yes	1.04 (0.58, 1.87)	0.878	0.98 (0.51, 1.85)	0.951	1.15 (0.67, 1.99)	0.600	1.12 (0.62, 2.04)	0.691
Adjuvant chemotherapy, yes	1.06 (0.70, 1.60)	0.752	1.04 (0.66, 1.63)	0.862	1.19 (0.81, 1.75)	0.36	1.27 (0.83, 1.94)	0.271
Intravesical instillation, yes	0.48 (0.31, 0.72)	0.000	0.46 (0.28, 0.73)	0.001	0.57 (0.39, 0.83)	0.003	0.53 (0.34, 0.82)	0.005
Surgery approach, LRNU	2.08 (1.38, 3.14)	0.000	2.07 (1.30, 3.29)	0.002	1.41 (0.97, 2.05)	0.068	2.00 (1.29, 3.11)	0.002
LVI, yes	2.89 (1.17, 7.14)	0.021	2.98 (1.20, 7.41)	0.018	2.42 (1.05, 5.59)	0.037	2.84 (1.15, 7.01)	0.023
Hydronephrosis, yes	1.42 (0.78, 2.58)	0.24	1.98 (0.97, 4.06)	0.059	1.66 (0.95, 2.88)	0.071	1.90 (0.96, 3.76)	0.065
Tumor necrosis, yes	2.01 (0.84, 4.78)	0.115	/	/	1.94 (0.86, 4.37)	0.106	2.02 (0.84, 4.86)	0.113
Multifocality, yes	1.68 (0.85, 3.30)	0.131	1.67 (0.79, 3.51)	0.177	1.80 (0.96, 3.39)	0.067	1.49 (0.74, 3.01)	0.257
Tumor location		0.227		0.282		0.973		0.452
Ureteral vs. Renal pelvis	1.32 (0.83, 2.10)	0.227	1.31 (0.77, 2.21)	0.314	1.04 ( 0.67, 1.59)	0.855	1.24 (0.75, 2.04)	0.392
Both vs. Renal pelvis	0.74 (0.33, 1.68)	0.484	0.70 (0.29, 1.69)	0.435	0.97 (0.46, 2.05)	0.946	0.80 (0.35, 1.83)	0.602
pT-stage		0.000		0.000		0.003		0.000
T2 vs. Tis Ta T1	2.34 (1.14, 4.79)	0.02	4.02 (1.45, 11.1)	0.007	2.12 (1.17, 3.82)	0.012	3.93 (1.55, 9.95)	0.004
T3-T4 vs. Tis Ta T1	4.40 (2.31, 8.39)	0.000	8.96 (3.52, 22.82)	0.000	2.54 (1.48, 4.35)	0.001	7.74 (3.26, 18.34)	0.000
pN-stage, N+	2.11 (0.84, 5.29)	0.109	1.96 (0.78, 4.94)	0.152	1.83 (0.77, 4.34)	0.171	1.74 (0.69, 4.36)	0.232

UTUC, upper tract urothelial carcinoma; RNU, radical nephroureterectomy; LVI, lymphatic vascular infiltration; LRNU, laparoscopic RNU.

**Figure 1:**
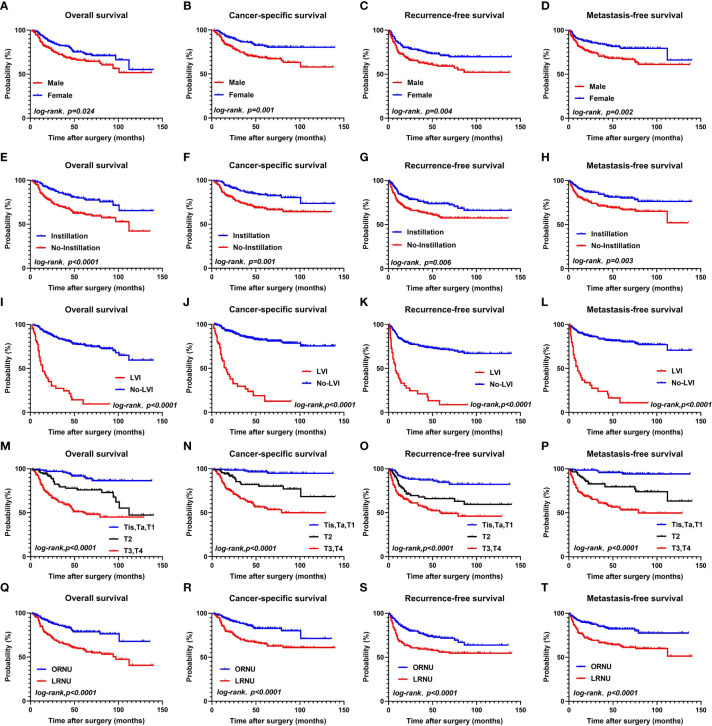
OS, CSS, RFS, MFS between the female group and male groups **(A–D)**; OS, CSS, RFS, MFS between the instillation group and the No-instillation groups **(E–H)**; OS, CSS, RFS, MFS between the LVI group and the No-LVI groups; **(I–L)**; OS, CSS, RFS, MFS between the Tis, Ta, T1 group and T2 group, T3-T4 group **(M–P)**; OS, CSS, RFS, MFS between the ORNU group and LRNU groups **(Q–T)**.

Next, we performed Kaplan–Meier analysis to assess the risk classification by sex, postoperative intravesical instillation, LVI, pT stage, and surgery type (**Figures 1A–T**, all P<0.05).

Finally, according to the multivariate Cox regression analysis results that identified the independent risk factors and the number of risk factors that each patient had, the 401 patients were divided into three risk groups: low-risk group (0 or 1 risk factors; 133 cases), medium-risk group (2 risk factors; 136 cases), and high-risk group (3–5 risk factors; 132 cases).

**Figure 2:**
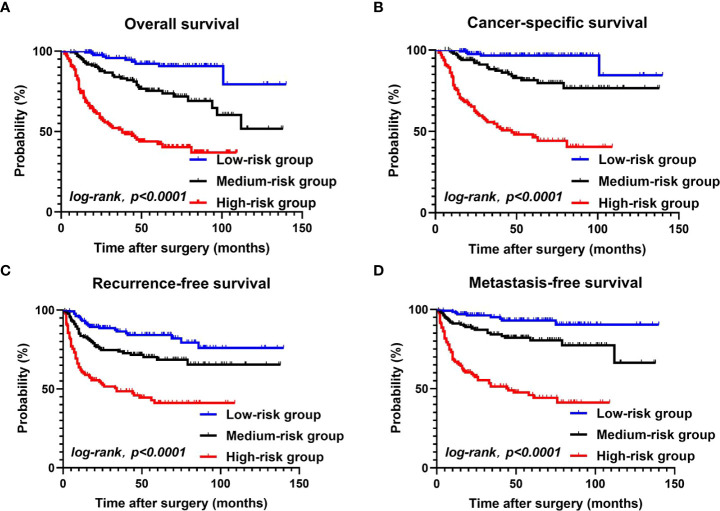
OS, CSS, RFS, MFS between the low-risk group, the medium-risk group, and the high-risk group **(A–D)**.

Kaplan–Meier curves of each group showed the following. Overall survival (OS) at 1, 3, and 5 years was 99.2%, 95.7%, and 90.7%, respectively, in the low-risk group. 95.6%, 83%, and 73.7%, respectively, in the medium-risk group, and 74.1%, 51.3%, and 42.3%, respectively, in the high-risk group. Cancer-specific survival (CSS) at 1, 3, and 5 years was 98.4%, 96.6%, and 84.5%, respectively, in the low-risk group, 97%, 87.3%, and 79.7%, respectively, in the medium-risk group, and 76.1%, 54.7%, and 46.4%, respectively, in the high-risk group. Recurrence-free survival (RFS) at 1, 3, and 5 years was 93.2%, 86.4%, and 82%, respectively, in the low-risk group, 83.6%, 73.7%, and 68.6%, respectively, in the medium-risk group, and 60.5%, 48.4%, and 41%, respectively, in the high-risk group. Metastasis free survival (MFS) at 1, 3, and 5 years was 97.7%, 95.1%, and 90.4%, respectively, in the low-risk group, 91.2%, 84.2%, and 80.5%, respectively, in the medium-risk group, and 66.8%, 51.4%, and 46%, respectively, in the high-risk group. (**Figures 2A–D**).

## Discussion

Prognostic factor analysis and risk stratification are cornerstones of cancer management. Clinicopathological prognostic factors can be used for risk stratification to help doctors to identify high-risk patients who may benefit from NAC and/or AC, low-risk patients who may benefit from a conservative approach (thereby reducing the risk of overtreatment), and medium-risk patients whose treatment plans can be similarly individualized. Currently, the main challenge faced by UTUC patients is the high recurrence and metastasis rates after RNU (16). It has been reported that the mortality rate was 21.9% and the recurrence rate was 25.4% in UTUC patients at 30 months after RNU (17). Similarly, our results showed that the mortality rate of UTUC was 22.7% and the recurrence rate was 32.4%. Therefore, it is extremely important to simultaneously predict OS, RFS, and MFS of UTUC patients after RNU based on clinicopathological prognostic factors.

Thus, we identified the key risk factors leading to poor prognosis among UTUC patients, and then established a risk stratification model for OS, CSS, RFS, and MFS based on the risk factors, in order to predict the prognosis of UTUC patients and thereby provide recommendations for AC and NAC use. After risk stratification, the 5-year OS, CSS, RFS, and MFS of the high-risk patients significantly lower than those of the other groups(P<0.0001). Our multivariate Cox regression analyses confirmed a number of variables that had previously been recommended by European Association of Urology (EAU) guidelines as prognostic factors (2, 5). More specifically, our analysis showed that being male, pT stage, LVI, and intravesical instillation were independent predictors of OS, CSS, RFS, and MFS among UTUC patients; intravesical instillation was a protective factor, while all the other factors were risk factors. Additionally, we concluded that LRNU was an independent risk factor for OS, CSS, and MFS among UTUC patients.

There are few and controversial studies on sex differences in UTUC prognosis (18), and the sex differences are influenced by regional and cultural factors. In the United States and Western European countries, the prevalence of UTUC is usually 1.5–2.5 times higher in men than in women (19–21). In Asian countries, the prevalence of UTUC is 1.3 times higher in women than in men (22–24). Being male is also a key predictor of adverse outcomes in UTUC patients in China, whereas univariate analysis showed that sex was not a key predictor in the United States (25, 26). One hypothesis for the sex difference in China is that women in some areas of China use traditional Chinese herbs containing aristolochic acid to treat certain basic diseases, and aristolochic acid has been reported to cause UTUC (27). Another hypothesis for the sex differences in UTUC is related to hormonal and anatomical differences between males and females, which have been suggested to affect the recurrence, progression, or CSS of bladder cancer (28) and are assumed to also relate to UTUC (which is similar in some ways to bladder cancer). However, this remains at the level of hypothesis and a clear theoretical background has not yet been established (29); the hypothesis needs to be verified in multi-regional and multi-center studies.

As a widely recognized prognostic factor, the prognostic value of LVI has been revealed in many urinary system tumors, such as prostate (30), bladder (31), and penile (32) cancer. In our study, the rate of LVI was 11.2%. The impact of LVI on prognosis was consistent with previous research, with LVI being a prognostic risk factor in UTUC (33). Of course, whether LNs are invaded is also an important prognostic factor, but we believe that LVI may be a better prognostic indicator than LN metastasis. Studies have shown that LVI is a prerequisite for LN metastasis of tumor cells, which enter into the blood *via* the microcirculation and eventually form micrometastases (34). LVI based on pathology results indicates a significant increase in the risk of metastasis (35). Therefore, it can be concluded that LVI plays an important role in metastasis, and LVI may become a key pathological finding after RNU or biopsy in the decision regarding whether to undergo treatment with NAC and/or AC (10, 36, 37). In contrast, the use of LN dissection (LND) in prognostic prediction is limited by the fact that there is no clear consensus on the scope and number of LN that should be dissected, though EAU guidelines recommend that LND should be based on anatomical templates (38). Moreover, it is often difficult to verify whether a patient’s outcome was contributed to by LND itself or by AC after surgery. Furthermore, LND may prolong the operation time, cause lymphatic leakage, and affect the postoperative recovery time of patients, so it is often difficult to achieve optimum LND. Nevertheless, a study reported that LND was a key factor affecting the prognosis of UTUC patients, and the number of LNs dissected was significantly correlated with CSS (39). Although the impact of LND on the survival rate is still controversial, we believe it is necessary to perform LND for patients.

We assessed the associations between surgical type and UTUC prognosis and found that LRNU was not associated with RFS (P=0.068) but was independently associated with poor OS, CSS, and MFS. Other research has reported that the oncological outcomes of LRNU and ORNU seem to be equivalent, including regarding OS, CSS, and RFS (40). However, LRNU was superior to ORNU in terms of perioperative outcomes, including decreased blood loss and shorter recovery or hospital stays (41). Although LRNU is an advanced technique, its feasibility in oncology remains controversial. In particular, a laparoscopic operation in a high-pressure intraperitoneal environment may increase the risk of tumor spillover (42). In addition, laparoscopic operation on larger tumors may lead to increased gravity-related migration of tumor cells and eventual implantation into the retroperitoneal space or bladder (43). Moreover, there may be insufficient LND during LRNU compared to ORNU. Furthermore, the management of the bladder cuff may lead to different oncological risks. For example, laparoscopic bladder cuff resection is somewhat technically challenging, which may increase the risk of postoperative urine overflow, and it is more likely that viable tumor cells will be left, increasing oncological risks (44). Nevertheless, as LRNU technology has evolved over the past decade, precautions recommended in UTUC treatment guidelines have increasingly been taken, such as using closed systems, avoiding tumor fragmentation, and promoting the use of inner pockets (45). Thus, the risk of tumor spread under the pneumoperitoneum has been significantly reduced. Overall, it is critical to adhere to strict oncology principles and increase proficiency in complex techniques to improve outcomes.

According to the NCCN guidelines, tumor staging is a key factor in UTUC management (46). Our results suggest that pT stage >pT2 is a risk factor for poor OS, CSS, RFS, and MFS in UTUC patients. In addition, high tumor stage has been reported to increase intravesical recurrence, and many patients with high pT stage have died from UTUC before developing intravesical recurrence (47). Urologists prefer intravesical instillation, especially multiple intravesical instillations, in these postoperative patients because of UTUC’s aggressivity and the risk of tumor cells spreading along the urinary tract. There is a general consensus that intravesical instillation, especially multiple intravesical instillations, should be provided to UTUC patients with advanced invasive tumor stage (pT2–T4) or high-grade tumors, but it may represent overtreatment for superficial (pTa–T1) or low-grade cases (48). In addition, although the POUT trial showed that patients with non-metastatic pT2–T4 N0–N3 UTUC after RNU who received AC had increased progression-free survival (36), cisplatin-based AC usually requires sufficient renal function and can cause permanent kidney damage. As most UTUC patients are elderly patients with poor renal function, NAC may be more suitable than AC. Currently, the NAC regimen for UTUC is derived from the NAC regimen for urothelial bladder carcinoma. The 2020 NCCN guidelines indicate that NAC may be considered for selected UTUC patients, especially those with high pT stage and/or high tumor grade. Several retrospective studies have reported that NAC can improve survival (10, 49). As we found remarkable differences in 5-year survival among patients in the high-, medium-, and low-risk groups, the treatment of patients in these different risk groups should be carefully considered. Patients in the high-risk group may have higher pT stage, tumor grade, and poorer prognosis, so they may benefit from NAC and/or AC. In the low-risk group, 5-year OS, CSS, RFS, and MFS were all >80%, so AC may be eschewed to avoid renal function damage. For medium-risk patients, comprehensive individualized treatment should be developed. We also expect that ongoing NAC phase II and III trials in UTUC patients will provide better guidance for patient treatment.

Our study has certain limitations. First, the RNU for patients in this study spanned a 10-year period and involved multiple surgeons, and the surgical expertise of the surgeons and the learning curve related to laparoscopic surgery may have varied. This study could not clearly determine whether these factors affected the survival outcomes. Second, this study is a single-center retrospective study with samples from a single province; it has a small sample size and a risk of bias. In the future, multicenter prospective studies with large sample sizes and long-term follow-up are needed to achieve more comprehensive results.

## Conclusions

Being male, LVI, pT stage >pT2, and intravesical instillation were independent predictors of OS, CSS, RFS, and MFS among UTUC patients. All were risk factors, except for intravesical instillation, which was a protective factor. Additionally, LRNU was an independent risk factor for OS, CSS, and MFS in UTUC patients. After risk stratification, the 5-year OS, CSS, RFS, and MFS of the high-risk group were 42.3%, 46.4%, 41%, and 46%, respectively. Patients in the high-risk group may benefit greatly from AC and/or NAC.

## Data availability statement

The original contributions presented in the study are included in the article/**supplementary material**. Further inquiries can be directed to the corresponding author.

## Ethics statements

The studies involving human participants were reviewed and approved by Harbin Medical University Cancer Hospital. Written informed consent for participation was not required for this study in accordance with the national legislation and the institutional requirements.

## Author contributions

JG and DD designed the study. JG and JinL collected the data. JG, JinL, JiaL, SL, and DD analyzed the data and drafted the manuscript. JG and DD revised and approved the final version of the manuscript. All authors contributed to the article and approved the submitted version.

## Funding

This research received no specific grant from any funding agency in the public, commercial, or not-for-profit sectors.

## Conflict of interest

The authors declare that the research was conducted in the absence of any commercial or financial relationships that could be construed as a potential conflict of interest.

## Publisher’s note

All claims expressed in this article are solely those of the authors and do not necessarily represent those of their affiliated organizations, or those of the publisher, the editors and the reviewers. Any product that may be evaluated in this article, or claim that may be made by its manufacturer, is not guaranteed or endorsed by the publisher.
